# Sperm selection by thermotaxis improves ICSI outcome in mice

**DOI:** 10.1038/s41598-018-21335-8

**Published:** 2018-02-13

**Authors:** Serafín Pérez-Cerezales, Ricardo Laguna-Barraza, Alejandro Chacón de Castro, María Jesús Sánchez-Calabuig, Esther Cano-Oliva, Francisco Javier de Castro-Pita, Luis Montoro-Buils, Eva Pericuesta, Raúl Fernández-González, Alfonso Gutiérrez-Adán

**Affiliations:** 1 Department of Animal Reproduction, INIA, Avda. Puerta de Hierro, Madrid, Spain; 2 Unit of Reproduction, Hospital Universitario Príncipe de Asturias, Alcalá de Henares, Spain

## Abstract

The ejaculate is a heterogeneous pool of spermatozoa containing only a small physiologically adequate subpopulation for fertilization. As there is no method to isolate this subpopulation, its specific characteristics are unknown. This is one of the main reasons why we lack effective tools to identify male infertility and for the low efficiency of assisted reproductive technologies. The aim of this study was to improve ICSI outcome by sperm selection through thermotaxis. Here we show that a specific subpopulation of mouse and human spermatozoa can be selected *in vitro* by thermotaxis and that this subpopulation is the one that enters the fallopian tube in mice. Further, we confirm that these selected spermatozoa in mice and humans show a much higher DNA integrity and lower chromatin compaction than unselected sperm, and in mice, they give rise to more and better embryos through intracytoplasmic sperm injection, doubling the number of successful pregnancies. Collectively, our results indicate that a high quality sperm subpopulation is selected *in vitro* by thermotaxis and that this subpopulation is also selected *in vivo* within the fallopian tube possibly by thermotaxis.

## Introduction

Before reaching the fertilization site, mammalian spermatozoa need to overcome a series of hurdles in the female genital tract^1,2^. After millions of spermatozoa are ejaculated into the vagina, the number reaching the oviduct is about six orders of magnitude lower^3^. In humans, this number is in the range of tens to hundreds^4^ while in other mammalian species, such as mouse, rat or pig, approximately one spermatozoon is associated with each cumulus-oocyte complex at the fertilization site^3^. Interestingly, these low sperm numbers are sufficient to seek out and fertilize the oocyte *in vivo*, while for successful *in vitro* fertilization, at least several thousands of ejaculated spermatozoa are required^1^. This discrepancy might be explained by different sperm populations within the ejaculate varying in maturity or intrinsic quality^5–7^. Further, only a fraction of spermatozoa from the ejaculate are apparently able to navigate the female genital tract to fertilize the egg^8^. Thus, it is widely accepted that there are strong selection pressures in the female genital tract that promote movement towards the fertilization site of a small subpopulation of spermatozoa of optimal quality^1^. In prior work, we observed selection of the sperm fraction with the greater DNA integrity in the mouse fallopian tube^9^. However, the features characterizing this optimal sperm fraction remain unknown, as does the selection process or mechanism involved. In effect, the lack of an effective sperm selection method *in vitro* for use in assisted reproductive technologies (ARTs) is thought to be one of the reasons for the relative low pregnancy rates achieved by procedures such as intracytoplasmic sperm injection (ICSI) in human clinical practice^10–12^. Hence, developing an *in vitro* sperm selection method that will effectively exclude spermatozoa with damaged DNA is one of the main challenges of ARTs, especially of ICSI^1,12–14^.

Several methods have been developed to prepare spermatozoa prior to ART in clinical practice^15,16^. Among these methods, swim-up and discontinuous density gradients (DG) are the most utilized in fertility clinics to select the fraction of motile spermatozoa^15^. Swim-up is based on gentle centrifugation of the semen and the subsequent recovery of those spermatozoa that swim away from the pellet into the fresh medium^15^. In contrast, the DG method is based on the capacity of motile spermatozoa to sediment in higher density medium when centrifuged within different layers of medium of different density^15^. Both techniques serve to recover a fraction of spermatozoa of higher quality than the initial pool in terms of factors including DNA integrity^17^, though the quality of the spermatozoa in terms of DNA damage and ROS production seems to be better for the swim-up procedure^18^. However, despite these effective sperm preparation methods, the efficiency of ICSI remains relatively low. A recent meta-analysis of IVF-ICSI cycles performed from 2008 to 2012 in US human fertility clinics (n = 369,382) indicated an implantation rate of 26% as the ICSI outcome when a male factor was present (n = 164,263) and 23% when there was no male factor (n = 205,119); percentages of miscarriage were 14.8% and 16.5% respectively^19^. To try to improve ICSI outcomes, new sperm processing methods have been tested in human fertility clinics. However, so far there has been little improvement. For example, sperm selection through magnetic activated cell sorting (MACS) has been shown not to improve implantation rates following ICSI^20^. Another promising technology, intracytoplasmic morphologically selected sperm injection (IMSI) has yielded conflicting results. Some authors report no improvement compared with conventional ICSI^21^ while others have obtained substantially improved embryo production and implantation rates (33% and 57% respectively with IMSI *vs* 19% and 27% respectively with ICSI, respectively)^22^. There is therefore still a need for new methods to select high quality spermatozoa for ICSI.

Within the mammalian female genital tract, at any given time only about 10% of spermatozoa in the ejaculate reach maturity through a process known as capacitation, which confers them the capacity to fertilize the oocyte^3^. This sperm fraction also has the capacity to follow migration signals up the fallopian tube towards the oocyte^23^. Thus, it seems that several sperm guidance mechanisms exist^23^, two of which act only upon capacitated spermatozoa: thermotaxis for long-range guidance in the fallopian tube^24^ and chemotaxis as a short-range guiding mechanism acting close to the oocyte within the ampulla^8^. Hence, both sperm thermotaxis and chemotaxis can be viewed as mechanisms for selecting capacitated and thus optimal spermatozoa to fertilize the oocyte.

Thermotaxis relies on a temperature gradient established within the fallopian tube as the consequence of a drop in temperature produced at the utero-tubal junction during ovulation^25–27^. Guided by this gradient, capacitated mammalian spermatozoa are able to swim away from the utero-tubal junction towards the warmer temperature where the oocyte awaits^23,24^. In this study, we examined the hypothesis of thermotaxis as an operative mechanism in mammals to select spermatozoa carrying genetic material of sufficient quality to support embryo development to term. Herein our goal was to improve ICSI outcome by sperm selection through thermotaxis.

## Results

### Sperm thermotaxis in mice and humans

**Figure 1:**
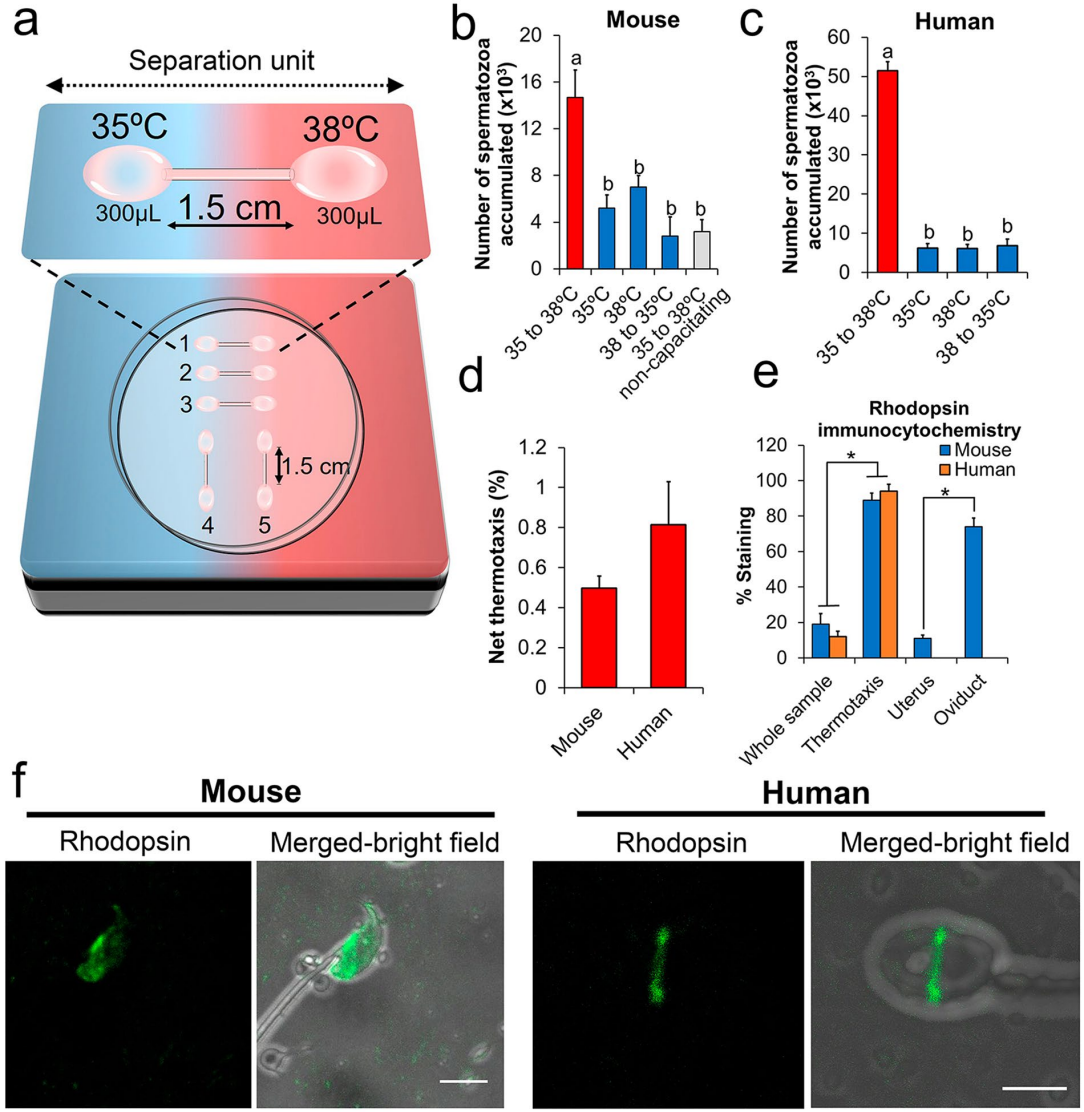
Spermatozoa selected by thermotaxis *in vitro*. (**a**) In the *in vitro* thermotaxis assay, spermatozoa are loaded in one drop of medium at one temperature and left to migrate to another drop at a higher temperature connected by a capillary tube (separation unit). See *text* for further details. (**b**) Number of mouse spermatozoa counted after migration across a temperature gradient or stable temperatures (mean ± SEM, n = 14, 1.5–3 × 10^6^ spermatozoa loaded per separation unit). Different letters indicate significant differences (*P* < 0.001 two–way ANOVA). (**c**) Number of human spermatozoa counted after migration across a temperature gradient or stable temperatures (mean ± SEM, n = 8, 3–10 × 10^6^ spermatozoa loaded per separation unit). *P < 0.001 two-way ANOVA. (**d**) Net thermotaxis of mouse and human spermatozoa (mean ± SEM, n = 14 and n = 8 respectively). (**e**) Percentage of spermatozoa showing specific rhodopsin staining in the unselected whole sample, in the subpopulation selected by thermotaxis, in spermatozoa flushed from the uterus, and in spermatozoa flushed from the fallopian tube (mean ± SEM; n = 5, 50–100 spermatozoa per determination). *P < 0.0001 two-tailed Student’s *t*-test. (**f**) Representative image of fluorescent immunocytochemistry showing the distribution of rhodopsin in mouse and human spermatozoa selected by thermotaxis. Bar = 5 µm.

Both mouse (F1 CBA/C57) and human spermatozoa were used in this study. We first designed a thermotaxis assay to assess sperm thermotaxis in these species based on detecting the transfer of spermatozoa through a capillary tube connecting two drops of medium (from now on referred to as separation unit) (Fig. 1a; see Supplementary Fig. S1 for a detailed description). Between these drops of medium a temperature gradient of 3 °C was set from 35 °C to 38 °C (temperatures were empirically determined in each of the drops with a thermocouple). This temperature gradient covers the range in which human and mouse spermatozoa respond to thermotaxis as reported elsewhere^28,29^. Further, it was chosen to cover the expected physiological gradient of temperature existing between the sperm reservoir and fertilization site in the oviduct *in vivo*, as measured in the pig^25^ and rabbit^26^. Spermatozoa were loaded in the drop at 35 °C and after 1 h those present in the drop at 38 °C were counted (separation units 1–3 in Fig. 1a). As controls, we also set up separation units with no temperature gradient (units 4 and 5 in Fig. 1a) or an inverse temperature gradient (38 °C to 35 °C) to identify random sperm movements. Before this test, mouse spermatozoa collected from the caudal epididymis were subjected to capacitating conditions for 30 min. Significantly higher counts were obtained using the gradient 35 to 38 °C than in the three controls (Fig. 1b), with no differences detected between controls. Further, spermatozoa processed under non-capacitating conditions failed to migrate from 35 to 38 °C (Fig. 1b) confirming the dependence of mouse spermatozoa on capacitation for their thermotaxis, as described in humans and rabbits^24,28^. Next, we prepared human ejaculates by swim-up for the thermotaxis test. Swim-up is one of the most widely used methods in fertility clinics to enrich sperm with high-quality spermatozoa before ART^15^. By subjecting the prepared spermatozoa to a temperature gradient from 35 to 38 °C for 1 h we confirmed human sperm thermotaxis, obtaining similar counts to those reported by others using different methods^28,29^ and lower counts in controls (Fig. 1c). The difference in spermatozoa counts between both species was due to the different quantity of spermatozoa loaded in each separation unit (~1 × 10^6^ mouse spermatozoa and 3–10 × 10^6^ human spermatozoa). Net thermotaxis rates (percentage of migrated spermatozoa referred to loaded spermatozoa) were higher in the human sperm samples (Fig. 1d).

### Rhodopsin distribution patterns in thermotactic spermatozoa

**Figure 2:**
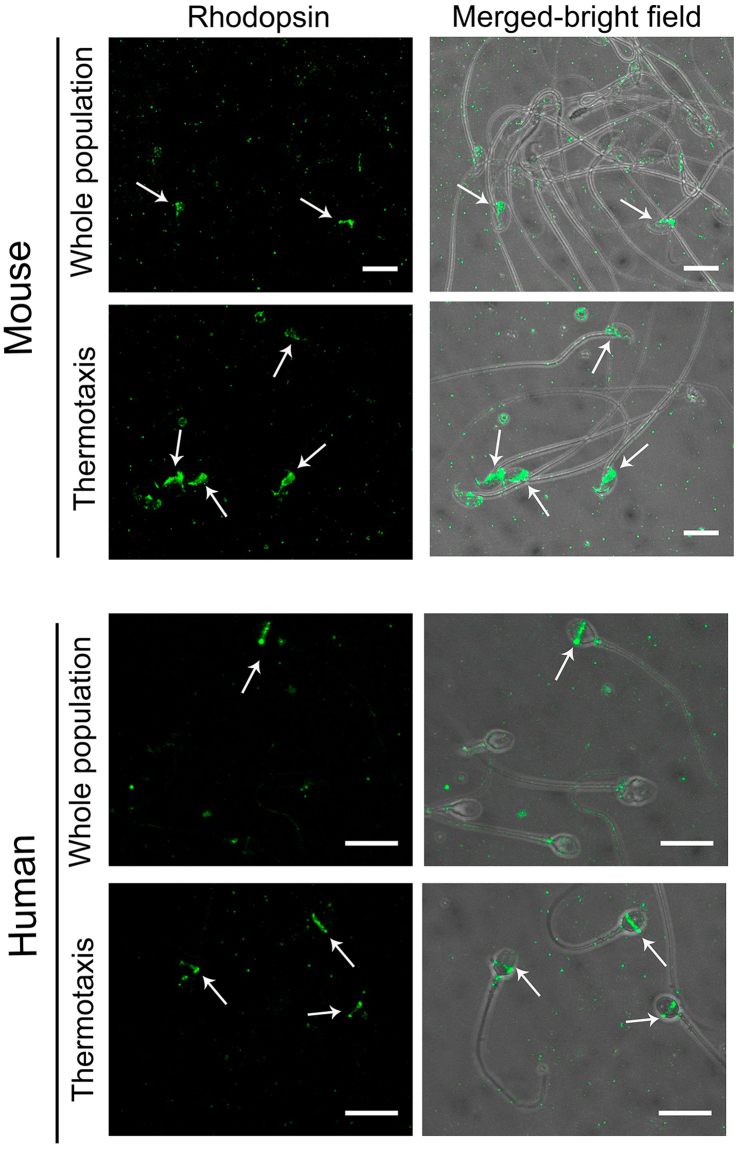
Fluorescence immunocytochemistry for rhodopsin. Mouse epididymal or ejaculated human spermatozoa immunostained for rhodopsin before selection (whole population) or after *in vitro* thermotaxis selection. Bars = 10 µm.

Opsins are a family of G-protein coupled receptors (GPCRs) thought to act as thermosensors for sperm thermotaxis^29^. Opsins show a heterogeneous distribution in the human ejaculate and in spermatozoa harvested from mouse epididymis, including different subpopulations according to the type of opsin present and its location^29^. Knocking out rhodopsin significantly reduces the thermotactic response of mouse spermatozoa^29^. Thus, we checked whether rhodopsin and/or its location could be used as a marker of the thermotactic sperm subpopulation. Rhodopsin was labeled by fluorescence immunocytochemistry employing a polyclonal antibody whose specificity against this opsin has been validated^29^. Remarkably, in both the mouse and human spermatozoa selected by thermotaxis using our *in vitro* system, rhodopsin appeared in a well-defined location (Fig. 1f), the equatorial segment of the head. However, in the mouse spermatozoon, rhodopsin appeared from the end of the acrosome to the neck, whereas in the human spermatozoon it formed a thin equatorial ring around the head. This staining pattern was observed in 19 ± 6% mouse spermatozoa retrieved from the epididymis and in 12 ± 3% human ejaculated spermatozoa respectively (n = 5; ~250 total spermatozoa) (Figs 1e and 2), similar to reported values^29^. In both species, this staining pattern was significantly enriched in the spermatozoa selected by thermotaxis to 89 ± 4% in mice (Figs 1e and 2) and to 94 ± 4% in human (n = 5; ~200 total spermatozoa) (Figs 1e and 2). This indicates that thermotaxis selects a specific subpopulation of spermatozoa carrying rhodopsin at a specific location and reinforces the hypothesis of rhodopsin as a thermosensor for sperm thermotaxis^29^. According to our data, rhodopsin staining also emerged as a marker of the thermotactic sperm subpopulation.

To examine whether the spermatozoa selected by thermotaxis were also selected *in vivo* in the fallopian tube, we immunocytochemically labeled rhodopsin in spermatozoa collected separately from the mouse uterus and fallopian tube. Individual crosses were programmed by natural mating, and 8 h later we euthanized the females to recover the spermatozoa by flushing the uterus and fallopian tube separately. Because of the low number of spermatozoa reaching the fallopian tube^3,4^, 6 crosses were conducted for each repetition and the flushed spermatozoa from the fallopian tube or uterus were separately mixed and concentrated by centrifugation (150 × g for 5 min). Then, through fluorescent immunocytochemistry, we were able to note the significant enrichment in the fallopian tube of spermatozoa showing rhodopsin at the location described above for the spermatozoa selected *in vitro* by thermotaxis (Fig. 1e and Supplementary Fig. S2): rhodopsin staining was 11 ± 2% (n = 5, 500 total spermatozoa) in the uterus versus 74 ± 5% (n = 5; 110 total spermatozoa) in the fallopian tube (Supplementary Fig. S2).

Our immunocytochemical experiments in the mouse revealed the heterogeneous location of rhodopsin in the unselected spermatozoa and the selection of a specific sperm subpopulation by thermotaxis *in vitro* that was consistent with the enrichment of the same subpopulation migrating to the fallopian tube *in vivo*. This suggests that only spermatozoa in which thermosensors (rhodopsin) are correctly located are able to migrate across the temperature gradient of the fallopian tube to reach the proximity of the oocyte.

### Quality of thermotactic spermatozoa

Previously, we reported that among the mouse spermatozoa that reach the uterus, only those with minimal amounts of fragmented DNA migrate towards the fertilization site in the fallopian tube^9^. Thus, it is currently accepted that within the fallopian tube a sperm subpopulation is selected according to its quality^1^. Thermotaxis is a good candidate mechanism for this selection because (i) only motile and capacitated spermatozoa respond to and migrate across a temperature gradient^24,28^, (ii) this long-range guidance mechanism may act at the level of the fallopian tube where selection occurs^8,9,23^, (iii) thermotaxis selects the same sperm subpopulation as that selected within the fallopian tube according to our immunocytochemical labeling of rhodopsin (Figs 1e, 2 and Supplementary Fig. S2). We then went on to determine whether thermotaxis *in vitro* selects a sperm subpopulation of characteristics considered quality markers of mammalian spermatozoa.

First, we examined sperm motion in the spermatozoa selected by thermotaxis and unseparated spermatozoa kept at 37 °C during thermotaxis (control). As expected, ~100% of both the mouse and human spermatozoa selected by thermotaxis were motile (Table 1). Using the computerized automatic sperm analysis system (CASA)^30^ at 37 °C, significant differences in kinetics were observed between the spermatozoa selected by thermotaxis and their controls in both mice and human samples (Table 1). Human spermatozoa selected by thermotaxis showed faster velocities, while the motility pattern in mouse spermatozoa selected by thermotaxis was less vigorous. However, although the kinetics reported here could reflect the motion characteristic of this subpopulation, alternatively we could be seeing an effect of the *in vitro* processing of the sample or of the migrating stimulus on sperm physiology. As reported in rabbit^24^ and humans^28^, and as we have observed in mouse, thermotaxis only occurred under capacitating conditions which has been shown to induce significant changes in sperm motility. This, together with our results, suggests specific motility modes characterizing the fraction of spermatozoa responsive via thermotaxis^31^. Further work is needed to address the physiological role of the observed kinetics. Notwithstanding, our results indicate the effective selection of motile spermatozoa and suggest a specific motility mode of this sperm subpopulation selected by thermotaxis.

**Table 1:** Kinetics of mouse and human spermatozoa unselected (control) or selected by thermotaxis.

Parameters CASA	Mouse (n = 14)		Human (n = 8)	
	Control	Selected	Control	Selected
VCL (µm s^−1^	169 ± 9	140 ± 5*	109 ± 4	122 ± 4*
VSL (µm s^−1^)	50 ± 5	39 ± 2*	52 ± 4	61 ± 3*
VAP (µm s^−1^	81 ± 4	77 ± 3	64 ± 3	71 ± 3*
LIN (%)	28 ± 2	29 ± 1	50 ± 4	51 ± 3
STR (%)	58 ± 4	51 ± 2*	76 ± 3	81 ± 2
WOB (%)	49 ± 1	57 ± 1*	62 ± 3	60 ± 3
ALH (µm)	8.1 ± 0.4	6.7 ± 0.3*	4.1 ± 0.3	4.5 ± 0.3
BCF (Hz)	5.4 ± 0.3	5 ± 0.1	10 ± 0.4	11 ± 0.2
MOT (%)	54 ± 9	100 ± 1*	65 ± 2	100 ± 1*

^*^P < 0.05 according to two-way ANOVA.

**Figure 3:**
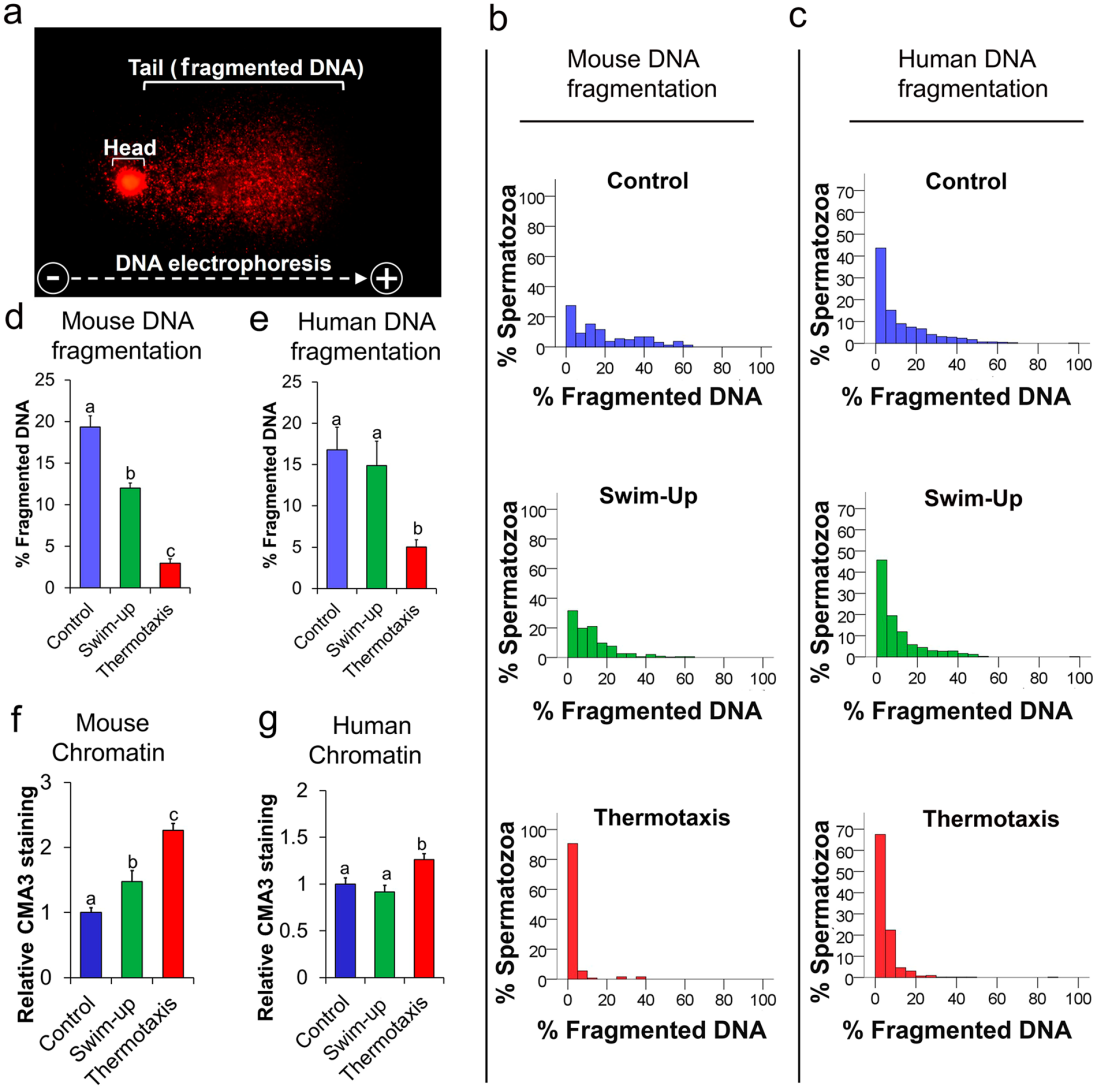
Quality of spermatozoa. Analyses performed in unseparated mouse and human spermatozoa (control –in humans this is the sample obtained after removing seminal plasma by centrifugation), after swim-up, or after selection by thermotaxis. (**a**) Representative image of SCGE in which fragmented DNA appears as a tail migrated from the nucleus (head) of the spermatozoa in response to electrophoresis. (**b** and **c**) Percentages of fragmented DNA determined using alkaline (mouse) or neutral (human) SCGE (mean ± SEM, n = 5 for mouse and n = 8 for human, 50–100 spermatozoa per determination). Different letters indicate significant differences (*P* < 0.001 two–way ANOVA). (**d** and **e**) Histograms showing the distributions of % of fragmented DNA in individual spermatozoa in each sample (n = 5 for mouse and n = 8 for human, 400–750 spermatozoa per histogram). (**f** and **g**) Chromatin compaction assessed through chromomycin A3 (CMA3) (n = 4, 50–100 spermatozoa per determination). Different letters indicate significant differences (*P* < 0.01 two–way ANOVA).

To assess the genetic quality of our spermatozoa selected by thermotaxis (both human and mouse), we examined DNA fragmentation and chromatin compaction levels compared to the control and swim-up separated spermatozoa. For DNA fragmentation we used the single cell gel electrophoresis assay (SCGE or comet assay) (Fig. 3a): the alkaline version for mouse spermatozoa and neutral version for human spermatozoa^32^. For chromatin compaction, the fluorophore chromomycin A3 (CMA3) was used, which stains DNA C–G rich sequences when accessible due to low chromatin compaction^33^. Through SCGE, both the human and mouse spermatozoa selected by thermotaxis were found to show significantly less fragmented DNA than both control and swim-up separated spermatozoa (Fig. 3b–e and Supplementary Fig. S3). Mouse swim-up separated spermatozoa showed less DNA fragmentation than controls, while human swim-up separated spermatozoa did not show this difference with control spermatozoa when results were averaged. However, swim-up separated spermatozoa from some of the human donors did feature less DNA fragmentation than controls, and spermatozoa selected by thermotaxis always showed the least DNA fragmentation (Supplementary Fig. S4). Furthermore, in both species we observed clear enrichment of the percentage of spermatozoa with scarce fragmented DNA in the spermatozoa selected by thermotaxis (Fig. 3b and c). Interestingly, both mouse and human spermatozoa selected by thermotaxis showed less chromatin compaction, revealed as a greater intensity of CMA3 staining compared to controls and swim-up separated spermatozoa (Fig. 3f,g and Supplementary Fig. S5). Human spermatozoa are reported to remodel their chromatin during capacitation and acrosome reaction *in vitro* reducing their chromatin compaction^34^. Thus, our CMA3 results could indicate the positive selection of capacitated spermatozoa characterized by lower chromatin compaction or that the thermotaxis stimulus plays a role in chromatin decondensation prior to fertilization. This chromatin remodeling affects its packaging and may help prepare chromatin for post-fertilization events^34^. For example, *in vitro* pre-treatment of bull spermatozoa to reduce chromatin compaction significantly improves embryo production by ICSI^35,36^. Our results reveal specific characteristics of the thermotactic sperm subpopulation including looser chromatin packaging and greater DNA integrity when compared to the whole unselected sperm population.

### Intracytoplasmic sperm injection (ICSI) using spermatozoa selected by thermotaxis

**Figure 4:**
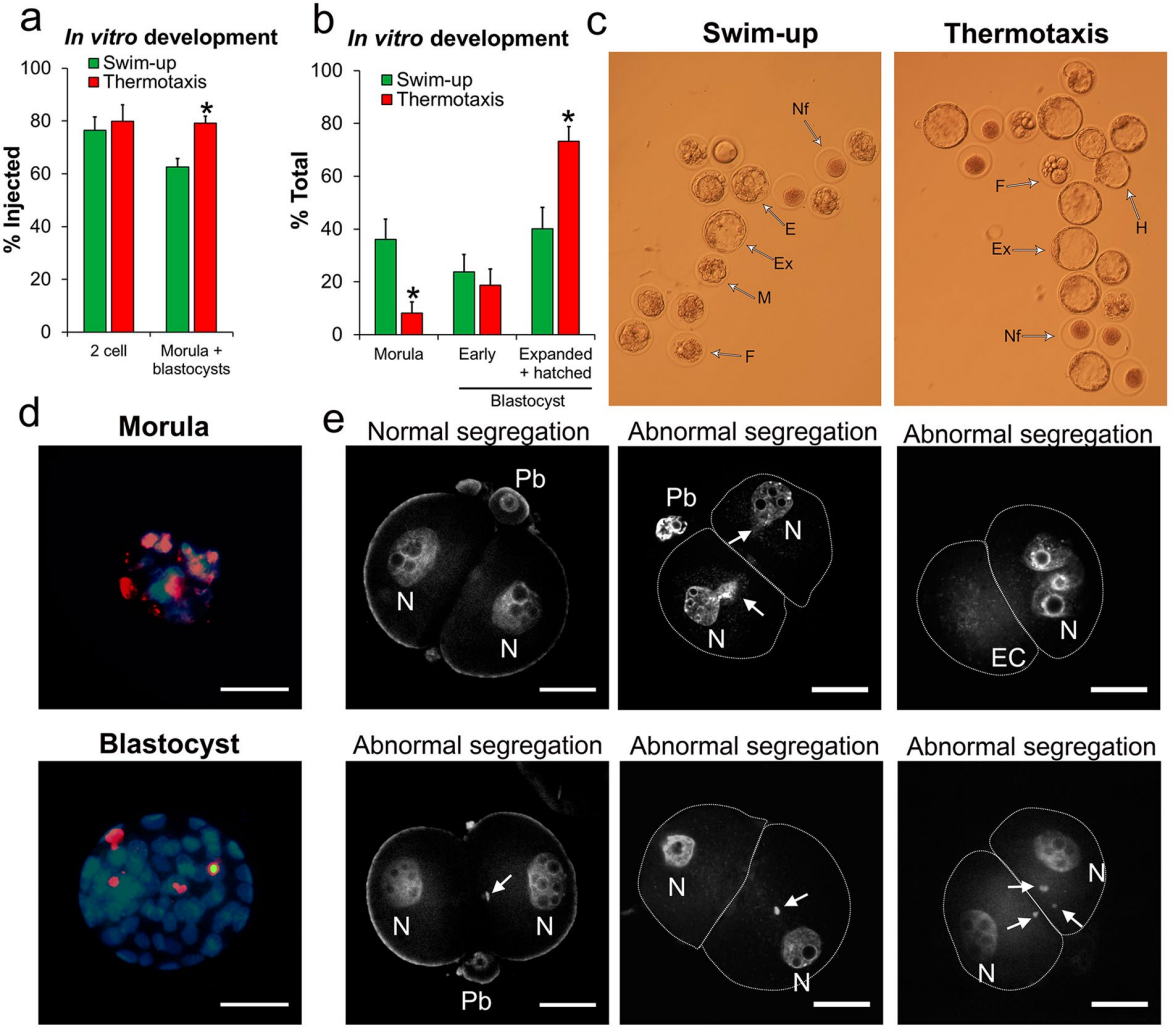
Quality of *in vitro* cultured mouse embryos produced by intracytoplasmic sperm injection (ICSI). For this experiment, swim-up separated *vs* thermotaxis selected spermatozoa were compared. (**a**) Morphological evaluation 24 h (% reaching 2-cell stage) and 5 days (% reaching morula + blastocyst stage) after ICSI (mean ± SEM, n = 8 experiments, 160 total ICSIs per sperm type). Different letters indicate significant differences (*P* < 0.01 two–way ANOVA). (**b**) Morphological evaluation 5 days after ICSI of morula and blastocyst embryos (mean ± SEM). Different letters indicate significant differences (*P* < 0.01 two–way ANOVA). (**c**) Representative image of embryos 5 days after ICSI cultured *in vitro*. Nf: not fertilized; F: fragmented/dead embryo; M: morula; E: early blastocyst; Ex: expanded blastocyst; H: hatching blastocyst. (**d**) Representative image of an *in vitro* cultured embryo processed for terminal deoxynucleotidyl transferase dUTP nick end labeling (TUNEL). Red fluorescence marks apoptotic/necrotic nuclei. Hoechst staining was used to counterstain nuclei (blue). Bar = 50 µm. (**e**) Representative images of 2-cell embryos stained with TO-PRO–3 Iodide for abnormal chromosome segregation assessment. Arrow: extranuclear DNA fragment; EC: empty cell; Pb: polar body; N: nuclei. Bar = 25 µm.

For intracytoplasmic sperm injection (ICSI), we used our mouse spermatozoa selected by thermotaxis and tested their capacity to support embryo development. We followed a standardized ICSI procedure for mouse^37^ and utilized swim-up separated spermatozoa as the control because swim-up is widely used for sperm quality enrichment in fertility clinics^15^. After ICSI, the embryos were cultured *in vitro* until the first division (2-cell embryos) or until the blastocyst stage before their examination (see below) or their transfer to pseudopregnant females, as described elsewhere^37,38^. Through this approach, we noted significant improvement in morula + blastocyst production using spermatozoa selected by thermotaxis (Fig. 4a) as well as a significantly higher proportion of expanded and hatched blastocysts compared to the use of swim-up separated spermatozoa (Fig. 4b and c). Using the TUNEL method, we compared apoptotic/necrotic cells numbers in the blastocysts^39^ and in both cases (swim-up separated spermatozoa or spermatozoa selected by thermotaxis) embryos arrested at the morula stage were all apoptotic/necrotic (see Fig. 4d for a representative image). Further, embryos that reached the blastocyst stage contained similarly low numbers of apoptotic cells in both groups, averaging 4 ± 0.8 and 4.1 ± 0.8 cells/embryo for embryos produced by ICSI using swim-up or thermotaxis selected spermatozoa respectively (mean ± SEM, n = 20 blastocysts/sperm group) (see Fig. 3d for a representative image). Blastocysts obtained by IVF (conducted in parallel as a control) showed 3.4 ± 0.3 apoptotic cells/embryo (mean ± SEM, n = 22 blastocysts), that is, comparable to the ICSI derived embryos (according to one–way ANOVA). These observations suggest that thermotaxis selects a subpopulation of mouse spermatozoa showing an improved ICSI outcome in that more viable embryos capable of full early development to blastocysts are generated.

ICSI can give rise to abnormal chromosome segregation during the first mitotic division observable as extranuclear DNA fragments at the 2-cell stage interphase^40,41^ (Fig. 4e). By staining 2-cell embryos with TO-PRO-3 Iodide (ThermoFisher scientific, MA, USA), we detected higher percentages of ICSI-derived embryos with these extranuclear DNA fragments when swim-up separated spermatozoa were used (11 ± 1%, n = 4, 78 total embryos) rather than thermotaxis separated spermatozoa (3 ± 2%, n = 4, 83 total embryos) (mean ± SEM; *P* = 0.03 two-tailed Student’s *t-*test) (see Fig. 4e for representative images). This abnormal segregation has been attributed to double-strand DNA breakage of the sperm-derived genome^40^. Our results support this hypothesis since spermatozoa selected by thermotaxis featuring less DNA fragmentation than swim-up separated spermatozoa also gave rise to 2-cell embryos displaying a lower incidence of abnormal segregation events. This abnormal segregation has been also suggested as the underlying reason for pregnancy loss following ICSI^40^ and could explain the difference observed here in blastocyst production using swim-up- or thermotaxis selected spermatozoa (Fig. 4a–c).

**Figure 5:**
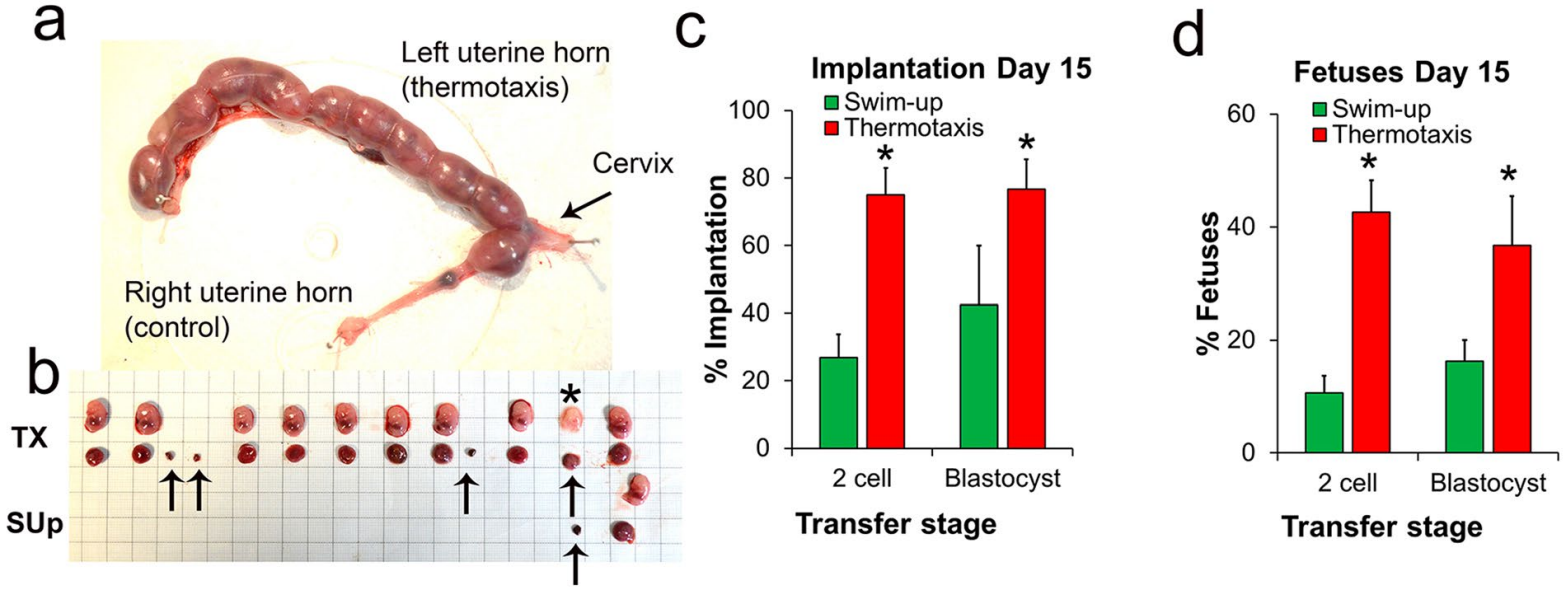
Transfer of ICSI produced mouse embryos. (**a**) Case of a mouse uterus showing the implantation on Day 15 post ICSI of 2-cell embryos transferred to a pseudopregnant female. Image shows 15 embryos derived from thermotaxis selected spermatozoa transferred to the left uterine horn and 15 embryos derived from swim-up separated spermatozoa transferred to the right uterine horn. (**b**) Fetuses (above) and their placentas (below) as well as reabsorptions (arrows) extracted from the uterus shown in panel a. Asterisk marks a late reabsorption. (**c**) Percentage of implantations on Day 15 referred to transferred embryos (mean ± SEM). (**d**) Percentage of fetuses on Day 15 referred to transferred embryos (mean ± SEM). (**b** and **d**) For the transfer of 2-cell embryos: n = 10, 15 embryos per transfer; and for the transfer of blastocysts: n = 4, 10 blastocysts per transfer; (*P < 0.05 Student’s *t*-test).

For a physiological quality assessment of our ICSI-produced embryos, we examined their implantation and development capacity *in vivo* by transferring them to pseudopregnant females. Two-cell embryos were transferred to the fallopian tube and blastocysts to the uterus^37,38^. To record implantation rates, surrogate females were euthanized 15 days after ICSI and implantations and fetuses counted (Fig. 5a–d). In both cases (2-cell embryos or blastocysts), significantly higher implantation rates were recorded for thermotaxis-derived than swim-up-derived embryos (Fig. 5c,d). Further, we also detected higher percentages of reabsorptions on day 15 post ICSI of transferred embryos derived from swim-up separated spermatozoa (60 ± 12% and 58 ± 8% for 2-cell embryos and blastocysts respectively) than from spermatozoa selected by thermotaxis (43 ± 5% and 46 ± 10% respectively) (Fig. 5a and b for a case showing much higher implantation and development of fetuses of transferred embryo derived from spermatozoa selected by thermotaxis than from swim-up separated spermatozoa). In the 2-cell embryo transfers (15 embryos per transfer), we also recorded higher rates of embryos developing to term for the spermatozoa selected by thermotaxis (5 and 13 pregnancies to term out of 24 transfers per group for swim-up–ICSI and thermotaxis–ICSI respectively) and higher rates of born pups for the thermotaxis-ICSI derived embryos (12 ± 2 and 29 ± 4% for swim-up- and thermotaxis separated spermatozoa respectively; *P* = 0.027 two-tailed Student’s *t* test). The newborn animals were morphologically normal and weight gain during the first three weeks was not different between groups as expected according to our previous work^37^. Late deleterious effects increasing body weight have been previously reported in ICSI-derived mice starting from week 9–11^37^. Accordingly, body weights recorded on day 270 after birth were significantly higher in our swim-up-ICSI derived pups (n = 10) than thermotaxis-ICSI derived pups (n = 20) (53 ± 2 and 41 ± 3 g respectively; *P* = 0.03 two-tailed Student’s *t*-test).

## Discussion

The results of our study suggest that thermotaxis acts as a selection mechanism while guiding spermatozoa through the fallopian tube. We observed here that only a fraction of spermatozoa bearing thermosensors is able to migrate within the fallopian tube, and that this sperm subpopulation selected *in vitro* by thermotaxis shows greater genetic integrity than the initial pool, as well as a lower level of chromatin packaging. Indeed, we observed in mice, that the use for ICSI on this selected sperm population improved the rate of correct zygote division into 2-cell embryos by reducing the abnormal chromosome segregation associated with this technique^40^. In addition, the high genetic quality of these selected spermatozoa also gave rise to improved rates of blastocyst production following their culture *in vitro* and of the implantation of 2-cell embryos and blastocysts transferred to surrogate females, significantly increasing the number of pregnancies to term and rates of born pups.

### Sperm heterogeneity and selection

Our findings link a sperm’s physiological characteristic –capacity to migrate in a temperature gradient– to the quality of its genetic content leading to the intriguing question of how is this link established. To answer this question, we should consider the reasons for the vast heterogeneity existing in any given sperm sample. First, numerous genetic combinations produced at meiosis generate a countless number of haplotypes^42^ determining that each spermatozoon is genetically unique. Further, as gamete production in the testis is constant and prolific, as a consequence errors accumulate in higher proportion than in the female germ line^43^. Accordingly, it has been suggested that the large numbers of spermatozoa in each ejaculate represents an evolutionary adaptation to offset inevitable errors in DNA replication during spermatogenesis and that most spermatozoa produced are incompetent for fertilization^44^. Although it is difficult to envisage to which extent the genetic variability generated in the testes, including all kinds of mutations, is behind sperm heterogeneity in the ejaculate, this variability explains the high percentage of spermatozoa of defective genetic content ejaculated. In humans it has been estimated that ~9% of spermatozoa within the ejaculate have chromosomal abnormalities^45^ and that each spermatozoon carries a number of *de novo* mutations^42^. This initial genetic variability might affect the performance of each spermatozoon, thus each haplotype may differentially affect spermatogenesis, producing different sperm subpopulations at the phenotypic level that are subsequently subjected to selection^46,47^. In agreement with this hypothesis, we found that the mice spermatozoa selected by thermotaxis led to a reduced incidence of chromosomal abnormalities after ICSI (Fig. 4d). Future studies need to address the incidence of chromosomal abnormalities directly in the sperm subpopulation selected by thermotaxis and to examine haplotypes by sequencing individual spermatozoa^42^. This would serve to determine whether specific haplotypes might confer differential properties at the physiological level. Although this innate genetic variance may have some impact on the performance of the different sperm subpopulations, other environmental factors are likely to show a stronger effect on individual spermatozoa generating the observed heterogeneity at the physiological level.

In this study, we observed high variability in DNA fragmentation levels shown by individual spermatozoa retrieved from the epididymis in mice (63% of the spermatozoa show a fragmentation higher than 5%, ranging from 5 to 65%) and by individual human spermatozoa present in the ejaculate of normozoospermic donors (56% of the spermatozoa showed fragmentation higher than 5%, ranging from 5 to 100%) (Fig. 3). The origin of this DNA fragmentation is multiple and may include: (i) the presence of different spermatozoa subpopulations in which physiological fragmentation occurring during the exchange of histones for protamines was not completely repaired^48^, (ii) aged and over-matured sperm subpopulations entering an apoptotic/necrotic process that generates DNA fragmentation^49^ and (iii) an effect of reactive oxygen species (ROS) production during the normal function of live spermatozoa^50^. The latter is a stressor produced by spermatozoa for diverse functions, including capacitation^49^.

We hypothesize that thermotaxis is a highly demanding mechanism that can only be undertaken by a small fraction of spermatozoa with intact cell structures. This means that most spermatozoa have damaged cellular components making them fail at different stages of their development prior to fertilization. Accordingly, we observed a large percentage of immotile spermatozoa among the sperm harvested from mouse epididymis and in the human ejaculate (Table 1, 46% and 35% respectively) which would be discarded by thermotaxis. Further, among the swim-up separated motile spermatozoa, we found different subpopulations showing relatively high DNA fragmentation. Indeed, DNA fragmentation in individual spermatozoa in the swim-up fraction did not differ from the proportions observed in unseparated spermatozoa (Fig. 3). This suggests a type of damage in most spermatozoa not linked to motility.

As thermotaxis involves relatively long distance migration along a tortuous fallopian tube, the machinery for this needs to be functional to operate to term. Thus, any minimal disruption will mean that the spermatozoon will not even get close to the egg. Effectively, thermotaxis only occurs under capacitating conditions^24,28^. This is also a highly demanding process involving many molecular changes that only ~10% of the spermatozoa are capable of at a given time point^51^. Thus, we would expect that a small sperm population within this 10% fraction of capacitated spermatozoa will also contain the correct machinery for successful thermotaxis-driven navigation. Accordingly, only less than 1% of both human and mouse spermatozoa were selected by our *in vitro* thermotaxis system. Further, we observed in both species that this small subpopulation showed rhodopsin labeling –indicating a sperm thermosensor^29^– at a specific location (Fig. 2). This subpopulation represented ~10% of unseparated human spermatozoa and ~20% of mice epididymal spermatozoa (Fig. 1e). This finding reflects the heterogeneity of the initial sperm sample and subsequent selection by thermotaxis of a small fraction in which the machinery for thermotaxis is correctly located. Interestingly, the same subpopulation was retrieved from the mouse fallopian tube after natural mating, supporting the hypothesis that thermotaxis operates as a selection mechanism within the fallopian tube (Supplementary Fig. S2).

### Implications for sperm studies

We here show that sperm selection by thermotaxis *in vitro* offers the possibility of examining an elite population of spermatozoa, which is likely to contain the spermatozoa responsible for fertilization *in vivo*. It should be noted that most sperm biology studies estimate average sperm parameters, while, as was commented above, most spermatozoa will be defective and will never have the potential to fertilize the egg *in vivo*. Thus, by analyzing thermotactic spermatozoa in detail we will have a more accurate understanding of sperm physiology and its contribution to the embryo. As also pointed out earlier, there is also a need to examine the genetics of individual spermatozoa selected by thermotaxis. Similarly, we should also examine other marks potentially contributing to fertilization and to subsequent embryo development. For instance, sperm samples are also heterogeneous regarding epigenetics as revealed by DNA methylation analysis of the human ejaculate^52^. These marks –including DNA methylation, histone modifications and RNA content^53^– should be analyzed in thermotactic spermatozoa to determine their role in early embryo development. Moreover, novel techniques currently available for single cell analysis could reveal different levels of heterogeneity in the selected subpopulation. Thus, the in depth study of this subpopulation could also help develop new tools to identify fertility markers and molecular targets, which are masked when averaged for the whole ejaculate, as candidates for novel sperm selection procedures^5^.

### Implications for ART

Most ARTs avoid the selection of spermatozoa occurring within the female genital tract. This could explain the low efficiency of both human^10–14^ and animal^37,54–56^ infertility treatments. Currently, ICSI is substituting IVF as the first option in human fertility treatment even when no male factor is present^19,57^. Given that infertile men subjected to fertility treatments show higher percentages of spermatozoa with fragmented DNA^13,58,59^, and that sperm DNA damage negatively affects clinical pregnancy following ART^60^, it has been stressed that the development of more selective methods for separating undamaged spermatozoa bearing high integrity DNA is essential to ensure the health of ART-derived individuals^1^. This is especially critical in the case of ICSI whereby all selective barriers for spermatozoa are bypassed, including the later barriers present in IVF such as cumulus penetration, membrane recognition and membrane fusion. Experiments in the laboratory mouse have revealed the risks and long term effects of ICSI on offspring including premature aging and a higher incidence of cancer, especially when sperm samples with damaged DNA were used^37,61^. Our results indicate that thermotaxis *in vitro* effectively selects a human sperm subpopulation showing scarce DNA damage. Further, using this selected sperm we dramatically improved ICSI outcome in the mouse, illustrating the potential of this technique for the selection of spermatozoa for ARTs.

## Conclusion

Our data point to thermotaxis as both a guidance mechanism and a mechanism of selecting high quality mammalian spermatozoa. In the mouse, its use dramatically improved the efficiency of ICSI giving rise to high quality embryos and thus improving the safety of the technique. Sperm thermotaxis has promising implications as a selection method for basic sperm studies and ARTs both in human and veterinary clinical practice.

## Methods

### Reagents and antibodies

All media components were purchased from Sigma–Aldrich (MO, USA) except where otherwise stated. The primary polyclonal antibody “rhodopsin (I-17)” and its blocking peptide as well as the secondary antibody “donkey anti-goat IgG-FITC” were purchased from Santa Cruz Biotechnology (TX, USA).

### Handling of mice and sperm samples

Experiments in mice were carried out in strict accordance with recommendations of the guidelines of European Community Council Directive 86/609/EEC. Every effort was made to minimize suffering. The study protocol was approved by the Committee on the Ethics of Animal Experiments of the INIA (2016 permit number CEEA 2014/025). Mice were euthanized by cervical dislocation. Spermatozoa were collected from the caudal epididymis of 3-month-old B6D2 males (resultant F1 from crosses between C57/BL/6 J males and DBA/2 J females) and suspended in modified human tubal fluid medium [HTF; 2.04 mM CaCl_2_ × 2H_2_O, 101.6 mM NaCl, 4.69 mM KCl, 0.37 mM KH_2_PO_4_, 0.2 mM MgSO_4_ × 7H_2_O, 21.4 mM sodium lactate, 0.33 mM sodium pyruvate, 2.78 mM glucose, 25 mM NaHCO_3_, 100 U/mL penicillin, 50 µg/mL streptomycin SO_4_ and 0.001% (w/v) phenol red] supplemented with 1% BSA. For capacitation, the samples were then incubated for 1 h under an atmosphere of 5% CO_2_ at 37 °C. When thermotaxis was tested under non-capacitating conditions, HTF was prepared without bicarbonate and the spermatozoa were incubated at 37 °C without CO_2_. HTF used for the thermotaxis test was supplemented with 0.025 mM of HEPES. In all cases, pH was adjusted to 7.4.

To recover spermatozoa from the uterus and fallopian tube, males were caged overnight, each with one female (CD-1) at estrus. Females with a vaginal plug were selected at 8 am the following day and the reproductive tract was carefully retrieved. Each fallopian tube was separated from the uterine horn by cutting at one third from the uterotubal junction. Each uterine horn was then separated by cutting its cervical end. Spermatozoa were flushed from the fallopian tube by injecting HTF through the infundibulum with the help of a syringe. The same was done for recovering spermatozoa from the uterus but injecting the HTF from the uterotubal-junction. In each experiment, 6 females were used and the spermatozoa from each structure were pooled.

### Mouse oocyte collection

Metaphase II oocytes were collected from the fallopian tubes of 6- to 8-wk-old female mice superovulated with 7.5 IU of eCG, and with an equivalent dose of hCG 48 h later. Briefly, at 14 h post-hCG administration, fallopian tubes were removed from superovulated female mice (C57BL/6 N) and placed in an M2-containing Petri dish at room temperature. After washing, collected fallopian tubes were placed in fresh M2 medium, and cumulus-oocyte complexes released from the ampulla with the aid of Dumont #55 forceps. Cumulus-oocyte complexes (COCs) were then transferred either into a fertilization drop (for *in vitro* fertilization [IVF]) or into a dispersion drop (for ICSI experiments). In ICSI experiments, cumulus cells were dispersed during 3- to 5-min incubation in M2 medium containing 350 IU/mL of hyaluronidase; after washing, oocytes were kept in KSOM medium at 37 °C in an atmosphere of 5% CO_2_ until use.

### Human sperm handling

Studies involving the use of human spermatozoa were approved by the ethics committee for clinic research of the Hospital Príncipe de Asturias, Madrid (code LIB 04/2016). Methods were carried out in accordance with approved guidelines. Human semen samples were obtained from healthy donors after 3 days of sexual abstinence. Informed consent was obtained from each donor. Semen samples showing normal density, sperm motility and morphology (according to WHO guidelines) were allowed to liquefy for 30–60 min at room temperature. Subsequently spermatozoa were separated from seminal plasma by centrifugation (240 × g, 10 min, twice) in capacitating medium (Global for Fertilization; LifeGlobal group, CT, USA) supplemented with additional human serum albumin (0.3% w/v final concentration). After the second centrifugation, the pellet was left for the spermatozoa to swim-up by the migration sedimentation technique^62^ in 1 mL of capacitating medium for 1 h under capacitating conditions of an atmosphere of 5% CO_2_ at 37 °C^51^.

### Thermotaxis assay

See Supplementary Fig. S1 for the thermotaxis assay set up and main text for conditions used during separation. To obtain the required number of spermatozoa for sperm analysis (except thermotaxis determination and motility) and ICSI, accumulated spermatozoa from 8 (mouse) or 4 (human) separation units (Fig. 1a) were pooled by centrifugation (300 × g for 5 min). The aliquots kept as controls (unseparated spermatozoa or swim-up separated spermatozoa) used for the same analyses or for ICSI were centrifuged as the test samples. Temperatures were empirically determined in each of the drops of the separation units with a thermocouple with an accuracy of ±0.5% of reading (T200KC, Radwell International, NJ, USA).

### Sperm motility

Mouse spermatozoa motility was examined by placing 20 µL of sperm suspension (2 × 10^6^ spermatozoa per mL) onto a pre-warmed slide on the stage heated to 37 °C of a Nikon Eclipse 50i microscope (Nikon, Tokyo, Japan) fitted with a digital Basler A312f camera (Basler AG, Ahrensburg, Germany) able to record 25 frames/s. For human spermatozoa motility, the same procedure was followed but placing 10 µL onto a pre-warmed slide and covering with a 22 × 22 mm coverslip. In each repetition, five 1 s videos (20–60 moving spermatozoa) were recorded in different fields and analyzed using the Integrated Semen Analysis System (ISAS; Projectes i ServeisR + D S.L., Valencia, Spain). In each experiment, three replicates were set up. The parameters analyzed were as described by Mortimer *et al*.^30^: straight-line velocity (VSL; time-averaged velocity of the sperm head along a straight line from its first position to its last position, expressed in µm/s); curvilinear velocity (VCL; time-averaged velocity of the sperm head along its actual curvilinear path, expressed in µm/s); average path velocity (VAP; velocity over an average path generated by a roaming average between frames, expressed in µm/s); linearity (LIN) (defined as (VSL/VCL) × 100); straightness (STR) (defined as (VSL/VAP) × 100); wobble (WOB) (defined as (VAP/VCL) × 100); amplitude of lateral head (ALH) displacement (width of the lateral movement of the sperm head, expressed in µm) and beatcross frequency (BCF; number of times the sperm head crosses the direction of movement per second, expressed in Hz).

### Immunocytochemistry

Mouse or human spermatozoa were washed in PBS by centrifugation (300 × g for 5 min) and adjusted to a concentration of 10^7^ spermatozoa per mL (as indicated above thermotactic spermatozoa were pooled from various separation units). Next, spermatozoa were smeared onto a glass microscope slide and left to dry. After three 5 min-washes in PBS, the slides were incubated overnight at 4 °C with the primary polyclonal antibody anti-rhodopsin I-17 diluted 1:50 in PBS containing 0.5% w/v of BSA. Following three washes in PBS, the slides were incubated for 2 h at room temperature with the secondary antibody diluted 1:50 in PBS. Subsequently the slides washed three times in PBS were mounted with Fluoromount aqueous mounting medium and examined in a fluorescence microscope.

### Single cell gel electrophoresis (SCGE)

Mouse spermatozoa were analyzed employing the alkaline version of the SCGE whereas for the human spermatozoa the neutral variant was used^32^. Microgels were prepared on slides precoated with 1% agarose by placing 85 µL of sperm suspension (2 × 10^6^) in 0.5% low melting point (LMP) agarose in PBS and then covered with a 22 × 22 mm coverslip. For gelation of the LMP agarose, the slides were left in a wet box at 4 °C for 2 h. After removing coverslips, slides were incubated at 37 °C for 1 h in lysis solution (2 M NaCl, 55 mM EDTA-Na_2_, 8 mM Tris, 4% Triton X-100, 0.1% SDS, 1 mM DTT and 0.5 mg/mL of proteinase K, pH 8). Next, the slides were washed twice in alkaline (0.3 M NaOH, 1 mM EDTA-Na_2_, pH 12) or neutral (90 mM Tris, 90 mM boric acid and 2 mM EDTA, pH 8.5) electrophoresis solution and then placed in the electrophoresis cell and filled with the same solution to cover the slides with about 1 cm of solution. Electrophoresis was run for 10 min at 25 v and the slides then neutralized in 0.4 M Tris-HCl pH 7.5 for 10 min. The slides were subsequently fixed in methanol for 3 min and air dried prior to staining with ethidium bromide for observation by fluorescence microscopy in a Nikon Optiphot-2 (Nikon, Japan). Comets were digitalized with a Nikon 5100 digital camera (Nikon, Japan) coupled to the microscope always under the same configuration in manual mode. Images were analyzed using the free software Komet casplab_1.2.3b2 (CaspLab.com)^63^.

### Chromomycin A3 (CMA3) staining

Spermatozoa at 2–5 × 10^6^ spermatozoa/mL were smeared onto microscope slides and left to dry. Slides were then washed twice in Mcllvaine solution (0.2 M Na_2_HPO_4_, 10 mM MgCl_2_ and 0.1 M acetic acid) and transferred to a wet box, covered with Mcllvaine solution, and incubated for 5 min at room temperature. Subsequently 100 µL of 0.25 mg/mL of CMA3 in Mcllvaine solution were added to each slide and the slides incubated in a wet box for 30 min in the dark. The slides were then washed twice in Mcllvaine solution for 5 min and mounted with Fluoromount aqueous mounting medium prior to their observation in a fluorescence microscope Nikon Optiphot-2 (Nikon, Japan). Images were captured with a Nikon 5100 digital camera (Nikon, Japan) coupled to the microscope always under the same configuration in manual mode. Photographs were taken by randomly moving across different fields on the slide. For each sperm sample, analysis was performed in triplicate.

### *In vitro* fertilization (IVF)

The method used has been described elsewhere^64^. Fertilization drops were prepared with 600 µL of HTF, covered with mineral oil and preincubated overnight at 37 °C in a 5% CO_2_ atmosphere. Oocytes were placed in groups of 30 to 50 COCs per drop. Then 10 µL of fresh epididymal sperm was added to the fertilization drop to achieve a final concentration of 1–2 × 10^6^ spermatozoa/mL and the drop incubated for 4 h at 37 °C in a humidified atmosphere of 5% CO_2_. Finally oocytes were cultured in KSOM as described below.

### Intracytoplasmic sperm injection in mice (ICSI)

Swim-up- and thermotaxis separated spermatozoa were pelleted in 0.2 mL tubes and frozen by immersion in liquid nitrogen. The frozen samples were stored for a maximum of one month at −80 °C until their use for ICSI. After thawing on ice, spermatozoa were resuspended in M2 medium. For the control, the spermatozoa were diluted to a concentration of 2.5 × 10^6^ spermatozoa/mL whereas the pellet of the spermatozoa selected by thermotaxis was resuspended in 10 µL of M2. A volume of frozen-thawed spermatozoa was mixed with 5 volumes of a 10% solution of polyvinyl-pyrrolidone (PVP; Mw 360,000) in M2 to give a final volume of 40–50 µL and placed on ice until use. ICSI was performed in M2 medium at room temperature. Individual sperm heads were injected into oocytes as groups of 20–40 oocytes. After a 15 min recovery period at room temperature in M2 medium, surviving oocytes were returned to EmbryoMax KSOM Embryo Culture medium (KSOM), covered with mineral oil and cultured at 37 °C in a 5% CO_2_ atmosphere.

### *In vitro* embryo culture

Embryos were cultured to the desired stage (2-cell or blastocysts) in groups of 20–40 embryo in 25 µL drops of KSOM, covered with mineral oil and placed in an incubator at 37 °C in a 5% CO_2_ atmosphere.

### Embryo transfer

Embryos were transferred into pseudopregnant CD1 mouse females mated with vasectomized mice as previously described^65^. The 2-cell embryos were transferred to the left fallopian tube 0.5 days post coitum (dpc), and the blastocysts were transferred to the left uterus 2.5 dpc.

### Terminal deoxynucleotidyl transferase dUTP nick end labeling (TUNEL)

Embryos at the morula or blastocyst stage were washed twice in 3 mL/mL PVP in PBS (PBS-PVP) and fixed in 4% paraformaldehyde in PBS for 15 min. After three washes in PBS-PVP, embryos were treated with 0.1% Triton X-100 in PBS for 30 min and washed twice again in PBS-PVP. Subsequently the TUNEL reaction was conducted using the *in situ* Cell Death Detection Kit (Roche, Switzerland) following the manufacturer’s instructions. Nuclei were counterstained with 0.1 mg/mL of Hoechst after three washes in PBS-PVP and mounted on a slide with Fluoromount aqueous mounting medium prior to fluorescence analyses.

### Determination of abnormal segregation during the first zygotic division

Two-cell embryos were washed in PBS-PVP and then fixed in 4% paraformaldehyde for 15 min. Embryos were then washed twice in PBS-PVP and stained with 0.1 mM of TO-PRO-3 Iodide (ThermoFisher scientific, MA, USA) and mounted on a slide with FluoroMount aqueous mounting medium prior to examination by confocal microscopy (Leica TCS-SP2, Leica, Germany).

## Electronic supplementary material

Supplementary Information
