# Supplementary material for: Sperm selection by thermotaxis improves ICSI outcome in mice
Source: Sci Rep. 2018 Feb 13;8:2902. doi: 10.1038/s41598-018-21335-8 (PMC5811574; doi:10.1038/s41598-018-21335-8)
Supplement: Supplementary file 1 — Supplementary Information [file 41598_2018_21335_MOESM1_ESM.pdf]

Supplementary Information for

Sperm selection by thermotaxis used to improve ICSI outcome

Serafín Pérez-Cerezales, Ricardo Laguna-Barraza, Alejandro Chacón de Castro, María Jesús Sánchez-Calabuig, Esther Cano-Oliva, Francisco Javier de Castro-Pita, Luis Montoro-Buils, Eva Pericuesta, Raúl Fernández-González, Alfonso Gutiérrez-Adán.

Correspondence to: s.perez.cerezales@gmail.com

This PDF file includes:

Figs. S1 to S5

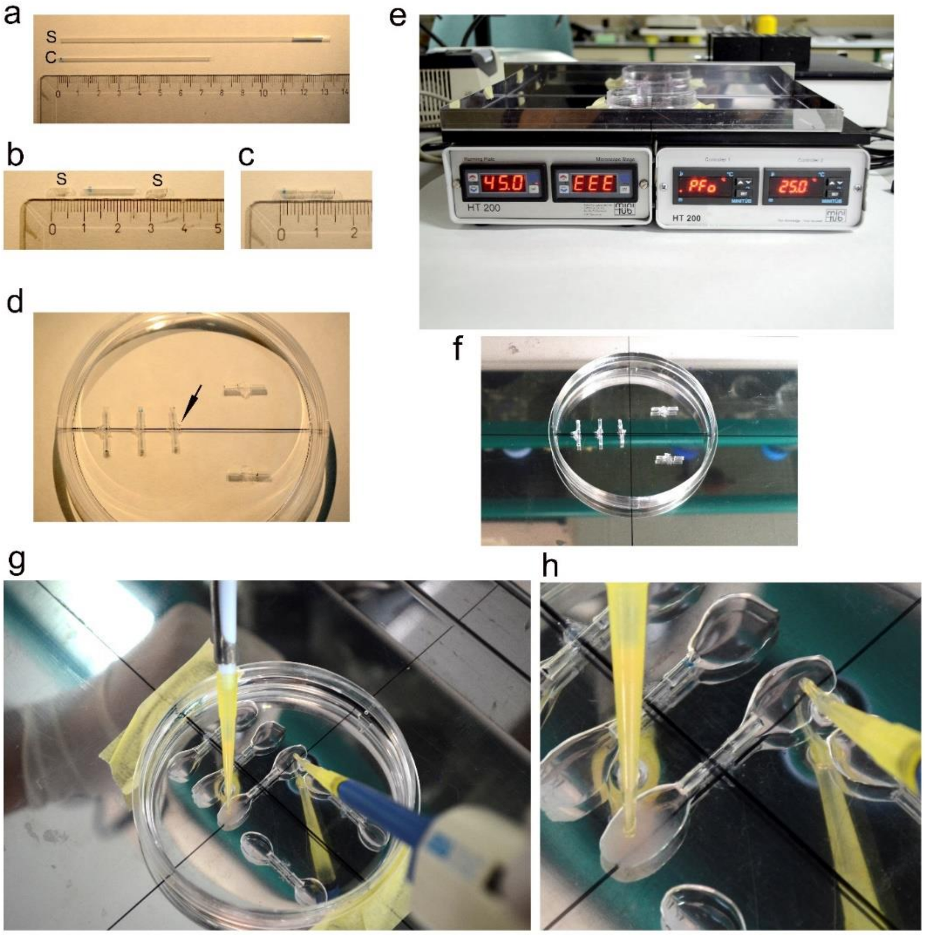

Fig. S1. *In vitro* thermotaxis system. (**a–c**) The separation units in Fig. 1. were prepared by cutting glass capillaries (**c**) of 1.15 ± 0.05 mm of internal diameter (Brand GMBH, Germany) into 1.5 cm lengths with the help of an abrasive file. Each end of these segments was introduced in a plastic end-piece of 0.5 cm made by cutting Mini straws (S) AI–2000 of 0.25 mL (IMV Technologies, France). (**d**) The assembled capillaries were placed on a cell culture Petri dish of 90 mm diameter (SPL Life Sciences, Korea) and attached in the middle with a drop of vaseline (arrow). (**e** and **f**) This mounted Petri dish was fixed with painter's tape onto an aluminum plate overlying two heating plates set at different temperatures to generate the desired temperature gradient. (**g-h**) Each capillary was then filled with capacitating or non-capacitating (depending on the experiment) medium supplemented with 0.025 mM of HEPES. Subsequently a 200-µL drop of this medium was placed at each end of the capillary such that the two drops were connected via the capillary duct. The Petri dish was then filled with mineral oil for mouse embryo and cell culture (Sigma-Aldrich, MO, USA) covering the drops completely and left for approximately 2 hours to equilibrate the temperature. The temperature between the two drops within a separation unit was empirically determined with a thermocouple (in our experiments 35 and 38ºC). Subsequently 100 µL of sperm suspension were loaded in one of the drops while at the same time 100 µL of medium were loaded in the opposite drop. After 1 hour, the two drops were disconnected by removing the capillary and accumulated spermatozoa were collected from the arrival drop for analysis. Sperm counting was performed with a hemocytometer.

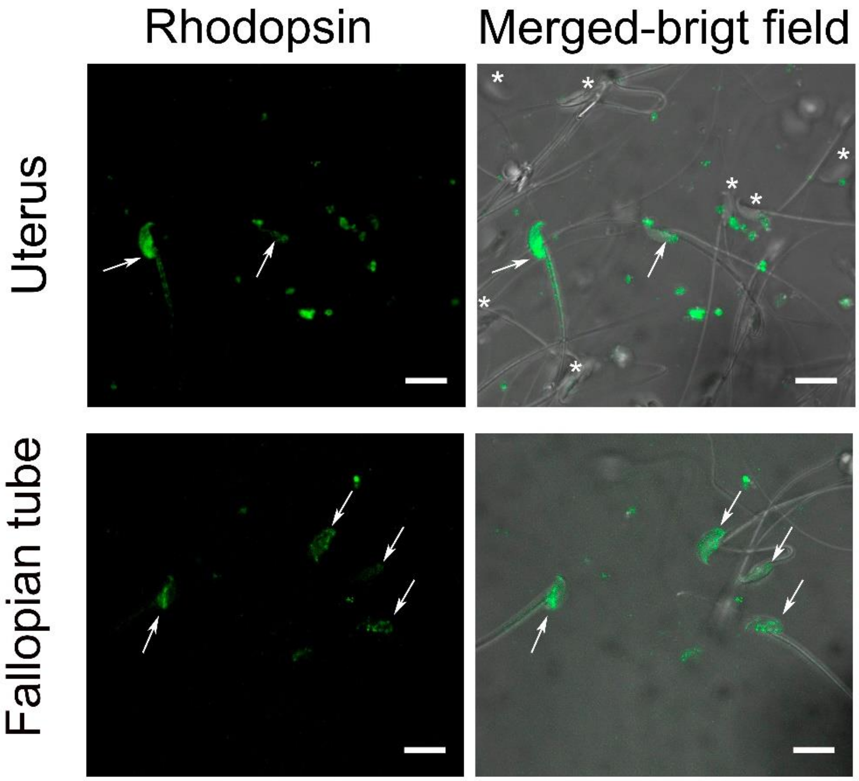

Fig. S2. Enrichment of mouse spermatozoa showing rhodopsin staining in the fallopian tube. Fluorescence immunohistochemistry for rhodopsin in spermatozoa retrieved from the uterus and fallopian tube. Arrows point to spermatozoa showing specific staining for rhodopsin. Asterisks in the merged bright field of the uterus indicate unstained spermatozoa. Bars = 10 µm.

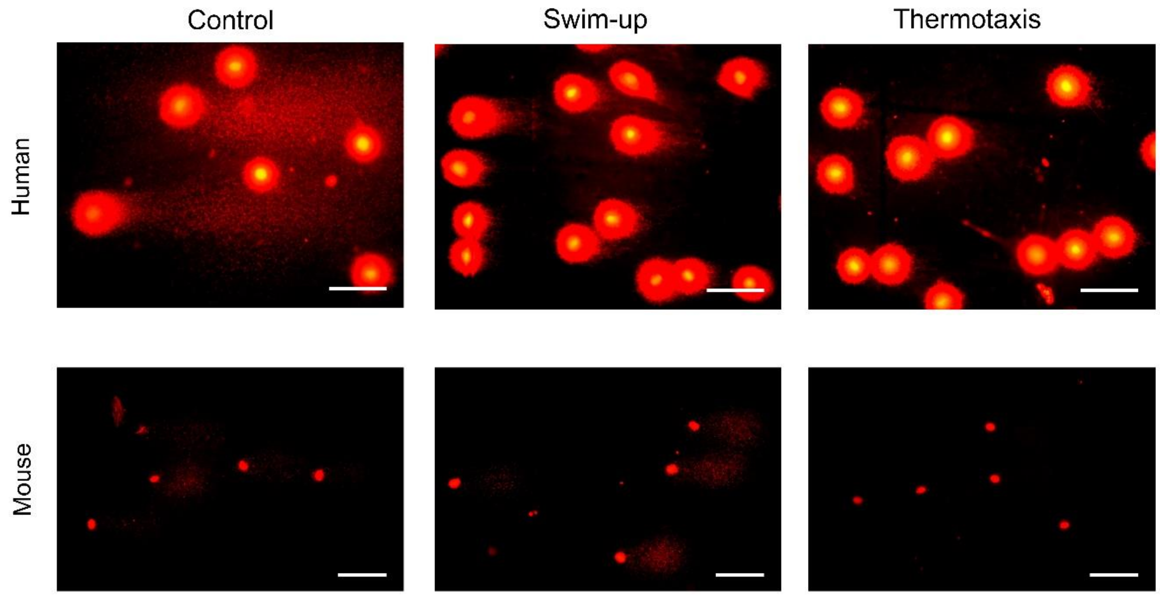

Fig. S3. SCGE analysis of human and mouse spermatozoa. Representative images of SCGE analysis of spermatozoa unseparated (control), separated by swim-up, or selected by thermotaxis. Images of human spermatozoa correspond to donor 7 in Fig. S4. Bars = 20 and 50 µm in humans and mice respectively.

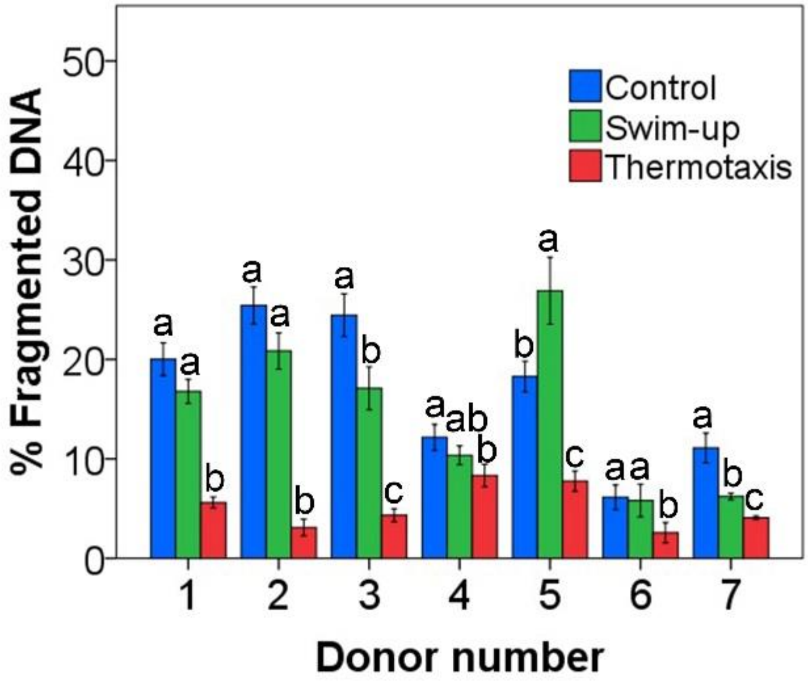

Fig. S4. SCGE analysis of spermatozoa from human donors. Percentage of fragmented DNA in spermatozoa unseparated (control), separated by swim-up or selected by thermotaxis in 7 donors. Different letters indicate significant differences ($P < 0.01$ two–way ANOVA).

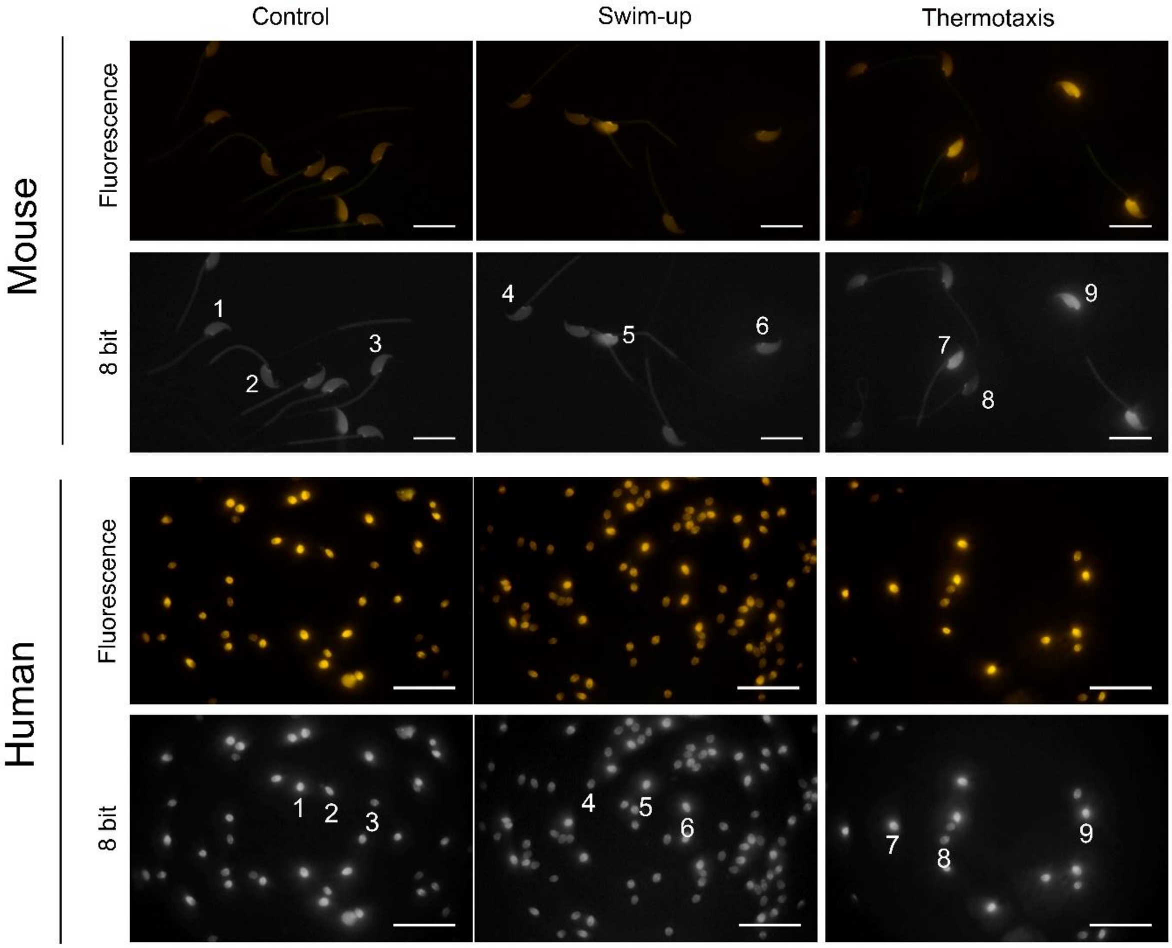

Fig. S5. CMA3 staining of mouse and human spermatozoa. Representative images of CMA3 staining of spermatozoa unseparated (control), separated by swim-up or selected by thermotaxis. For their analysis, the images were processed with ImageJ. Images were transformed to 8 bit and then each cell selected and measured for mean fluorescence intensity (arbitrary units). For example in the images (cell number=fluorescence intensity): 1=32; 2=33; 3=37; 4=27; 5=72; 6=41; 7=94; 8=23; 9=105 (mouse); and 1=142; 2=111; 3=61; 4=50; 5=117; 6=111; 7=126; 8=66; 9=139 (human). Bars = 13 µm for mouse and 30 µm for human spermatozoa.
